# Dynamic Tomographic Phase Microscopy by Double Six-Pack Holography

**DOI:** 10.1021/acsphotonics.1c01804

**Published:** 2022-04-08

**Authors:** Simcha K. Mirsky, Itay Barnea, Natan T. Shaked

**Affiliations:** Department of Biomedical Engineering, Faculty of Engineering, Tel Aviv University, Tel Aviv 69978, Israel

**Keywords:** digital holography, optical diffraction tomography, quantitative phase imaging, label-free microscopy, holographic multiplexing, 3D imaging

## Abstract

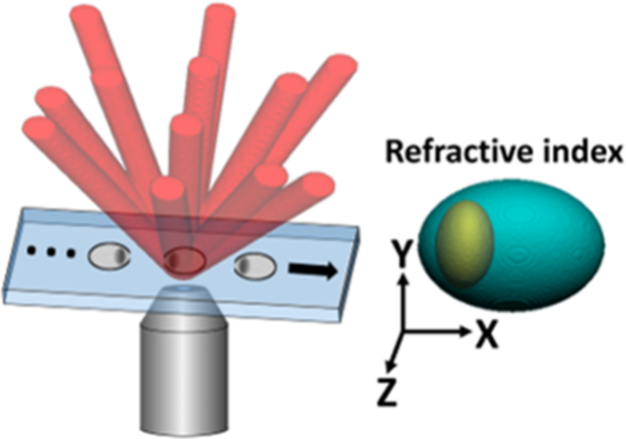

Three-dimensional (3D) optical imaging of rapidly moving biological cells is difficult to achieve as such samples cannot be scanned over time. Here, we present a dynamic scan-free optical tomography approach for stain-free 3D imaging of biological cells using our new double six-pack tomography technique, whereby 12 off-axis holograms are captured in a single camera exposure without sacrificing resolution or field of view. The proposed system illuminates the sample from 12 angles simultaneously, and 3D refractive index (RI) tomograms are reconstructed from each recorded video frame of the dynamic sample. The technique is verified experimentally by recording flowing silica beads, 3 μm in diameter, with the resulting tomogram RI accuracy being 98.5%. A live swimming sperm cell is also imaged, and dynamic 3D imaging results for both beads and sperm cell are presented. The proposed technique represents a 12-fold increase in dynamic holographic data for tomography.

## Introduction

In imaging flow cytometry, thousands of cells are imaged per second.^1^ However, the valuable three-dimensional (3D) morphology of these cells is not typically captured as it is difficult to accurately scan such rapidly moving samples in order to reconstruct a tomographic 3D model. Yet, this 3D data contains valuable information pertaining to the cell phenotype, such as the metastatic potential of cancer cells.^2^ Furthermore, as tomographic imaging tools become more readily available,^3,4^ there is increasing demand for improved tools to enable the 3D study of not only fast flowing cells but also dynamic living cells. The technique presented in this paper is aimed toward the eventual goal of making high-accuracy dynamic tomography a reality.

Until recently, holographic tomography was limited to static samples due to the need to rotate either the sample^5−7^ or the illumination^8−12^ or to scan multiple wavelengths^13^ and thus acquire the perspective images sequentially. Recent techniques have utilized either induced or natural rotation of samples during flow to acquire the perspective images over time.^14,15^ Additionally, techniques implementing rapid scanning have enabled acquisition of tomograms of dynamic samples,^10,12,16^ and multiplexing techniques have further pushed the boundaries of tomogram acquisition speed and synchronicity to the point of reconstructing tomograms from single camera exposures or video frames,^17−19^ resulting in dynamic tomography. However, the tomographic multiplexing techniques implemented until now have typically utilized standard off-axis holography setups and thus do not take full advantage of the camera spatial bandwidth, thereby limiting the amount of perspective image data that can be acquired per video frame. In this paper, we describe the first implementation of double six-pack holography and demonstrate its ability to dynamically acquire 12 times more perspective image data than previous techniques.

Holographic tomography is a technique for the reconstruction of the 3D refractive index (RI) distribution of a sample. Similarly to the computerized X-ray tomography used in medicine, many perspective images of the sample must be acquired from different angles, and then an algorithm is utilized to reconstruct the 3D distribution from the numerous two-dimensional (2D) images. While in X-ray tomography the 3D absorption distribution is reconstructed, in holographic tomography the complex images acquired enable reconstruction of the 3D RI distribution of the sample. These RI values correspond to different materials within the sample, such as cytoplasm, DNA, or protein.^20^

**Figure 1:**
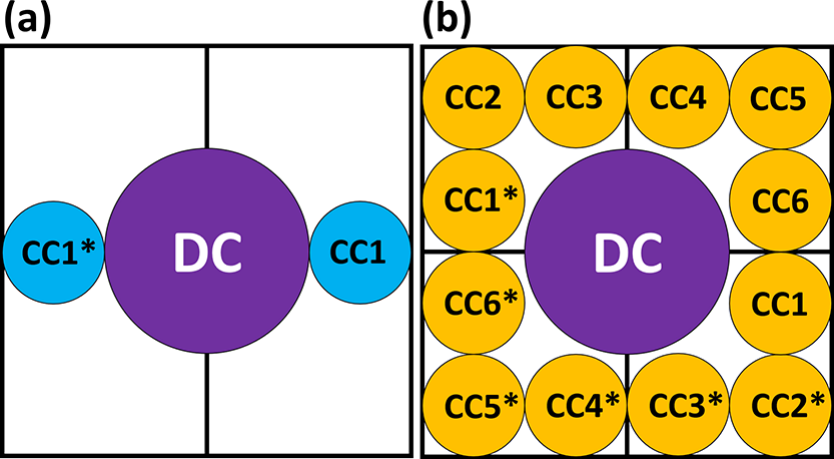
Illustration comparing the spatial frequency domain of six-pack holography to standard off-axis holography. (a) Standard off-axis holography, in which there is only one CC term and a matching complex conjugate CC term, shown as CC1 and CC1*, respectively. (b) Six-pack holography, in which there are six CC terms and matching complex conjugate CC terms, resulting in six times as much data per camera exposure as (a). CC, cross-correlation term; CC*, corresponding complex conjugate to CC of the same number; DC, autocorrelation term.

Six-pack holography is a technique developed by our group and previously demonstrated for applications in dynamic super-resolution imaging^21^ and out-of-focus light rejection^22^ as well as rapid phase reconstruction by digital compression.^23^ This technique takes advantage of the empty space in the spatial frequency domain of standard off-axis holograms, shown in Figure 1a, by multiplexing six pairs of cross-correlation (CC) terms in the spatial frequency domain without overlapping with the autocorrelation (DC) term, as shown in Figure 1b. In the hologram domain, this six-channel multiplexing is done by overlaying six off-axis interference patterns into the same hologram, but with different off-axis interference angles. This results in different fringe orientations and thus the six non-overlapping pairs of CC terms in the spatial frequency domain of the multiplexed hologram shown in Figure 1b. Six-pack holography achieves this by using six pairs of sample and reference beams, that is, a total of 12 beams, with the reference beam angles on the camera arranged precisely around the optical axis in order to place the CC terms in a square pattern. The problem of undesired interference between the non-matching beams is solved by using a low-coherence laser source and echelons to add different phase delays to each sample and reference beam pair, thereby preventing them from interfering with other beams—a technique known as coherence gating.^24^ The result is that six holographic images can be captured in the same camera exposure, without sacrificing image resolution or field of view and without requiring sample sparsity. Additionally, the CC term size may be decreased using a non-telecentric setup as proposed by Sánchez-Ortiga et al.^25^ By using a non-telecentric setup, two more holograms could potentially be multiplexed at the potential expense of aberrations due to field curvature.

**Figure 2:**
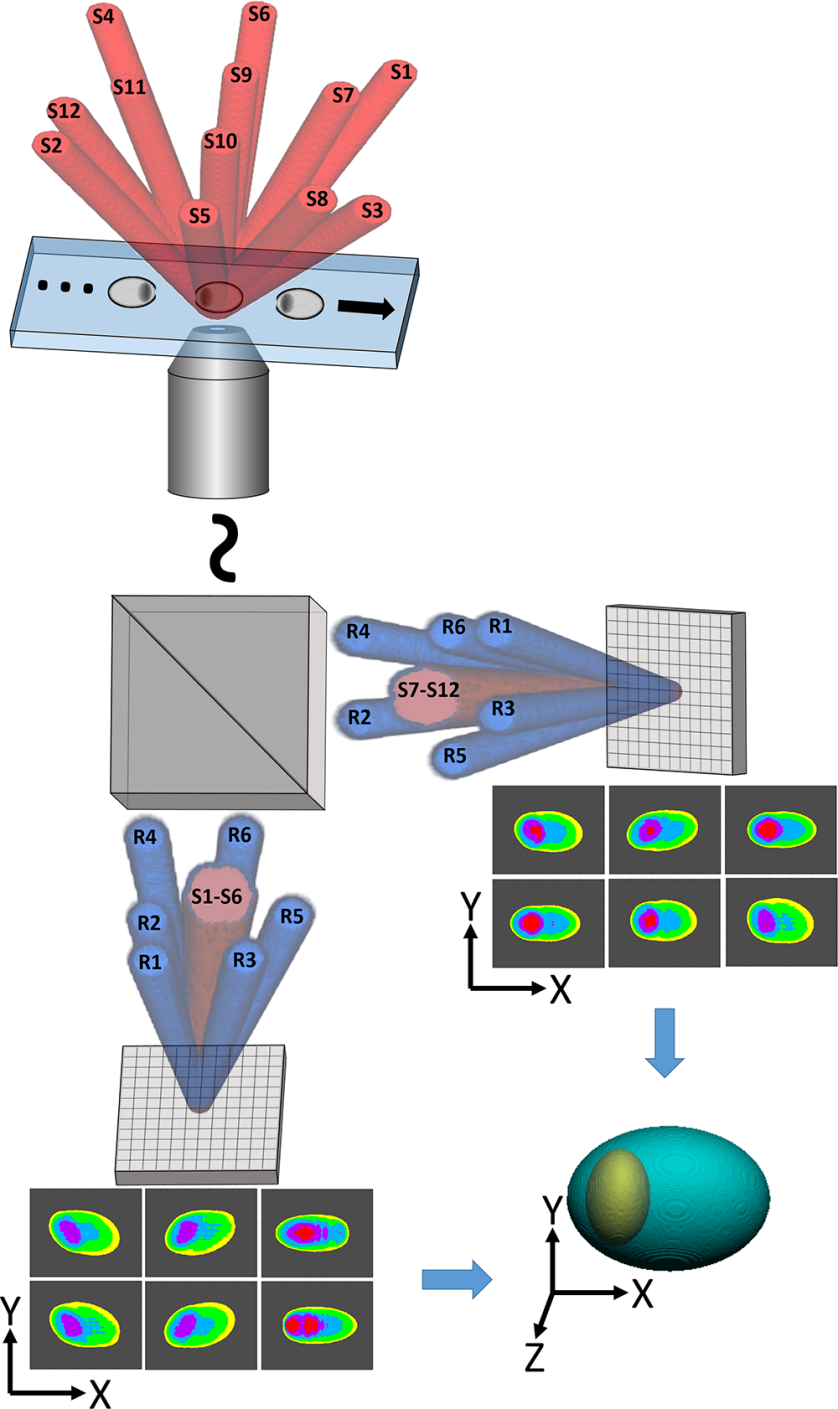
Simplified illustration of the double six-pack tomography system for 3D stain-free imaging of rapidly flowing biological cells. S1–S12, sample beams, illuminating the sample simultaneously; R1–R6, reference beams, interfering with the sample beams at the two detector arrays on which the holographic perspective images of the cell are recorded, from which the dynamic 3D RI profile of the sample is reconstructed.

In this paper, we present double six-pack holography. This method enables the acquisition of two different six-pack holograms simultaneously for a total of 12 perspective images, as illustrated in Figure 2. This is achieved by polarizing one set of six sample beams (S1–S6) orthogonally to a second set of six sample beams (S7–S12). Once the two sets are orthogonally polarized, they illuminate the sample from 12 different angles, and the two sets are imaged by the microscope using two cameras, each placed at one exit of the system. Polarizers are placed before each camera such that each camera receives only one set of sample beams, thereby preventing unwanted interference between sample beam sets. Six holographic perspective images, i.e., topographic phase maps, are reconstructed from the six-pack holograms of each camera, and these 12 holographic perspective images are then used to reconstruct the 3D RI tomograms from each video frame.

The proposed tomographic approach presents significant advantages with respect to the current state of art, in which sample rotation is induced and perspective images of the same sample are acquired sequentially over time.^14,15^ First, in the proposed technique, there is no need for samples to rotate as all perspective images are acquired simultaneously. This provides three benefits: the sample rotation no longer needs to be rigorously calculated in each video frame, the sample rotation does not need to be carefully controlled, and samples may be flowed faster as only a single multiplexed image is necessary for reconstruction. Finally, the reconstruction in the proposed technique is less sensitive to internal changes of the sample over time as only a single time point is acquired. Additionally, the proposed technique may be combined with the current sample rotation technique in order to increase reconstruction resolution. Aside from concerns of rotation, all the above advantages also apply when comparing the proposed technique to rapid scanning techniques,^12^ which may be more sensitive to changes in the sample over time and require reduced flow speeds in order to acquire the necessary number of perspective images.

## Results

In order to test the system on a sample with a well-known structure, silica beads 3 μm in diameter were mixed into a 10% polyvinylpyrrolidone (PVP, Sigma #PVP360) and 90% water solution, and 10 μL of the solution were placed on a standard glass slide, then covered by a #1 glass coverslip, and sealed with epoxy to prevent evaporation. Prior to the experiment, the RI of the solution was measured using a refractometer (ATAGO, PAL-RI) and was found to be 1.344.

**Figure 3:**
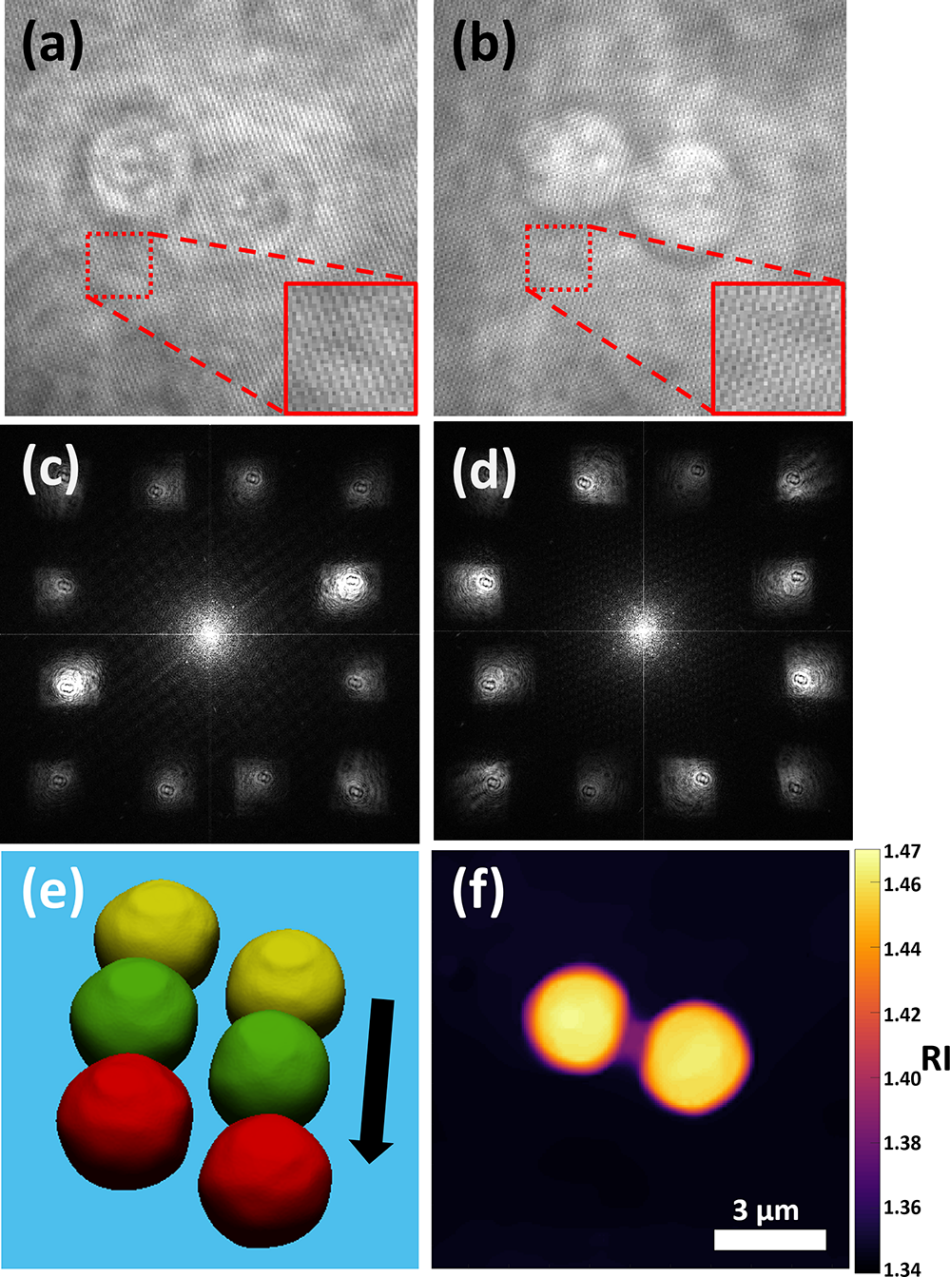
Double six-pack RI tomography results of 3 μm silica beads from a single video frame. (a,b) Two six-pack multiplexed off-axis holograms from cameras C1 and C2, respectively, where (b) is shown after flipping horizontally. (c,d) Spatial frequency power spectra of (a,b), respectively. (e) Overlays of isosurface renderings from the dynamic 3D RI tomogram reconstructed from the first frame (yellow), reconstructed from the middle frame shown in (a,b) (green), and reconstructed from the last frame (red) during microbead flow, shown at an oblique angle, with a surface isovalue of 1.40. The arrow indicates the direction of flow. (f) RI value map at the central plane. Scale bar of (f) also applies to (a,b).

A pair of beads was then recorded during flow, with the synchronized holograms, shown in Figure 3a,b, obtained simultaneously from a single video frame from each of the cameras, respectively. The spatial frequency power spectra of the holograms in Figure 3a,b are shown in Figure 3c,d, respectively, where it can be clearly seen that both holograms possess the six CC terms of six-pack holography. The rectangular shape of the CC terms may be due to the echelon composed of rectangular sections located in the conjugate Fourier plane. This did not present any significant adverse effects in the final phase images after division by the background image. Twelve phase map images were produced from the 12 CC terms, and the spatial frequencies of these images were mapped into a 3D spatial frequency domain based on their illumination angles, under the Rytov approximation. An inverse Fourier transform was then used to reconstruct the 3D RI tomogram.^26^ Isosurface renderings of three tomograms reconstructed from the 12 CC terms of three different video frames are shown in Figure 3e at an oblique angle, with a surface isovalue of 1.40. From this figure, it can be seen that both beads are reconstructed properly as spheres in each frame despite their proximity. The 3D shape of this tomogram, from a single video frame, is shown from multiple angles in Visualization 1, and a video tomogram of these flowing beads from two different viewing angles is shown in Visualization 2 at 200 fps. In this visualization, it can be seen that the 3D shapes remain consistent during the entire video, that is, 450 frames. Figure 3f shows the RI values of the beads from Figure 3e at the central plane using the perceptually uniform inferno color map,^27^ with the average RI of the entire section of beads being 1.449, which is 99% of the expected theoretical value of 1.457 for this wavelength^28,29^ or 93% relative to the RI of the medium. The average RI for the entire 3D structure of the pair of beads was found to possess a slightly lower value of 1.435, which is 98.5% of the expected theoretical value for this wavelength or 80% relative to the RI of the medium.

**Figure 4:**
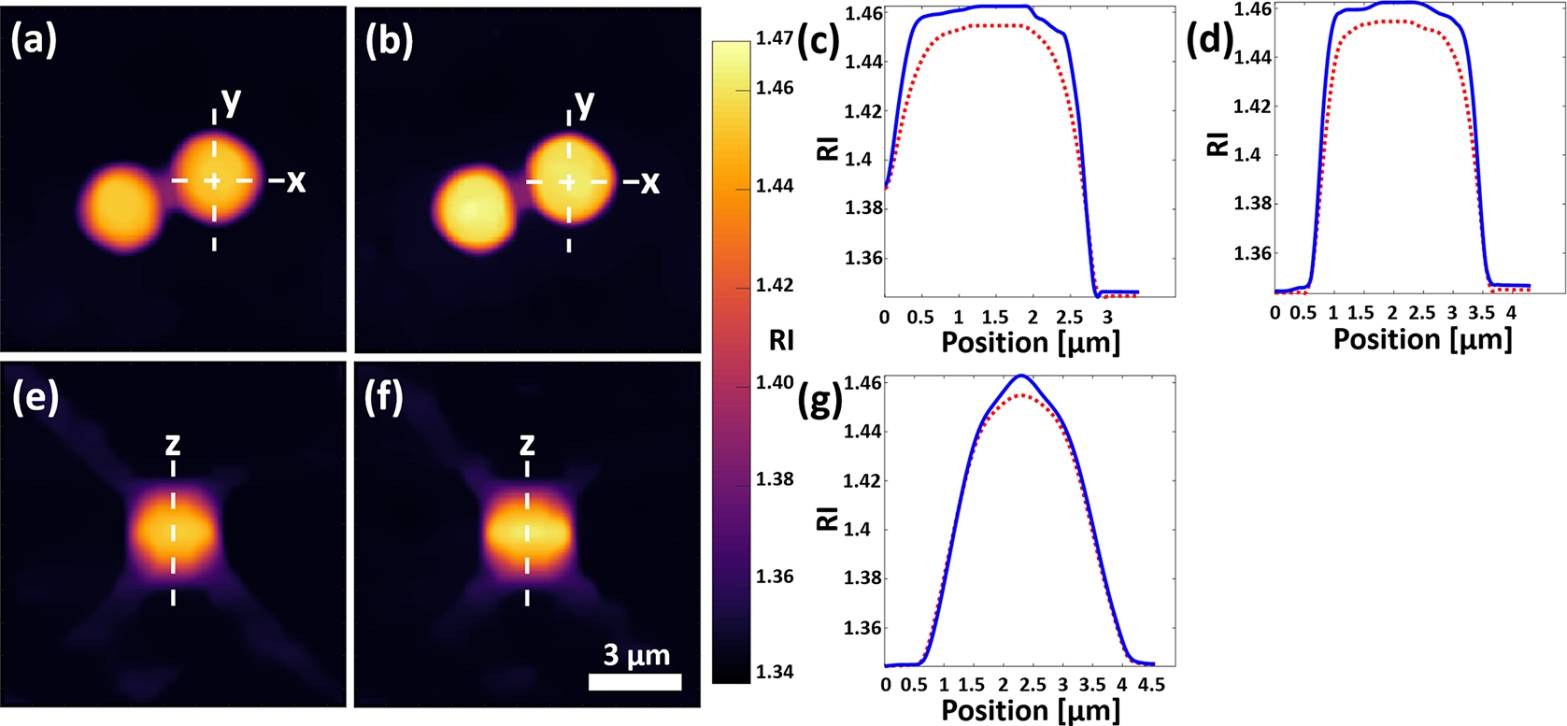
Cross-sectional images and RI profile curves of single and double six-pack tomograms of a bead pair. (a) Single six-pack tomogram RI map at the focal plane. (b) Double six-pack tomogram RI map at the focal plane. (c) RI profile curves along line *x* in (a,b). The red dashed line indicates the single six-pack tomogram. The blue line indicates the double six-pack tomogram. (d) RI profile curves along line *y* in (a,b). (e) Single six-pack tomogram RI values in the *yz* plane. (f) Double six-pack tomogram RI values in the *yz* plane. (g) RI profile curves along line *z* in (a,b). Scale bar in (f) applies to (a,b,e) as well.

In order to determine the resolution limits of our tomograms, we used the method proposed by Choi et al.^8^ on the RI tomogram of a bead, resulting in estimated spatial resolutions of 0.91 μm in the transverse *x* direction, 0.80 μm in the transverse *y* direction, and 2.23 μm in the longitudinal *z* direction. To determine the relative improvement in resolution of our double six-pack method versus that of a single six-pack hologram, we used the same method to determine the resolution limits from the RI tomogram of the same bead reconstructed from only sample beams S1–S6. The resulting estimated spatial resolutions of single six-pack holography were significantly larger in the transverse directions, with spatial resolutions of 1.40 μm in the *x* direction and 1.43 μm in the *y* direction. Thus, double six-pack holography improves the transverse resolution, reducing it by approximately 40%. The longitudinal *z* resolution of the single six-pack tomogram was not as significantly affected, with a resulting estimated value of 2.33 μm, indicating that double six-pack holography reduces this resolution limit only by approximately 4%. The RI maps and RI profiles used for the above calculations are shown in Figure 4.

Finally, we calculated the average RI of the single six-pack tomogram of the pair of beads at the focal plane and across the entire 3D structure, resulting in values of 1.435 and 1.425, respectively. These values are 98.5 and 97.8% of the expected theoretical value or 80.5 and 71.7% relative to the RI of the medium, respectively. Thus, the double six-pack tomogram provides RI values approximately 8% closer to the expected RI value relative to the RI of the medium when compared to the single six-pack tomogram.

Following this, human sperm cells were obtained from a donor and imaged. The study was approved by Tel Aviv University’s institutional review board. The sample was prepared as follows: the raw semen was incubated at room temperature for 1 h and then sperm cells were isolated using the PureCeption Bi-layer kit (ORIGIO #ART-2024), as per manufacturer’s instructions. Specifically, the semen was placed above a gradient of silica beads and centrifuged such that intact sperm cells concentrated in the pellet. The supernatant was removed, and the cells were washed with Quinn’s Advantage Sperm Washing Medium (ORIGIO #ART-1006) for 5 min at 1500 RPM. The resulting supernatant of intact sperm cells was then resuspended in 1 mL of 10% PVP and Quinn’s Advantage Sperm Washing Medium solution, and 10 μL of the final solution were placed on a standard glass slide, then covered by a #1 glass coverslip, and sealed with epoxy. The RI of the medium was measured using a refractometer and found to be 1.351.

**Figure 5:**
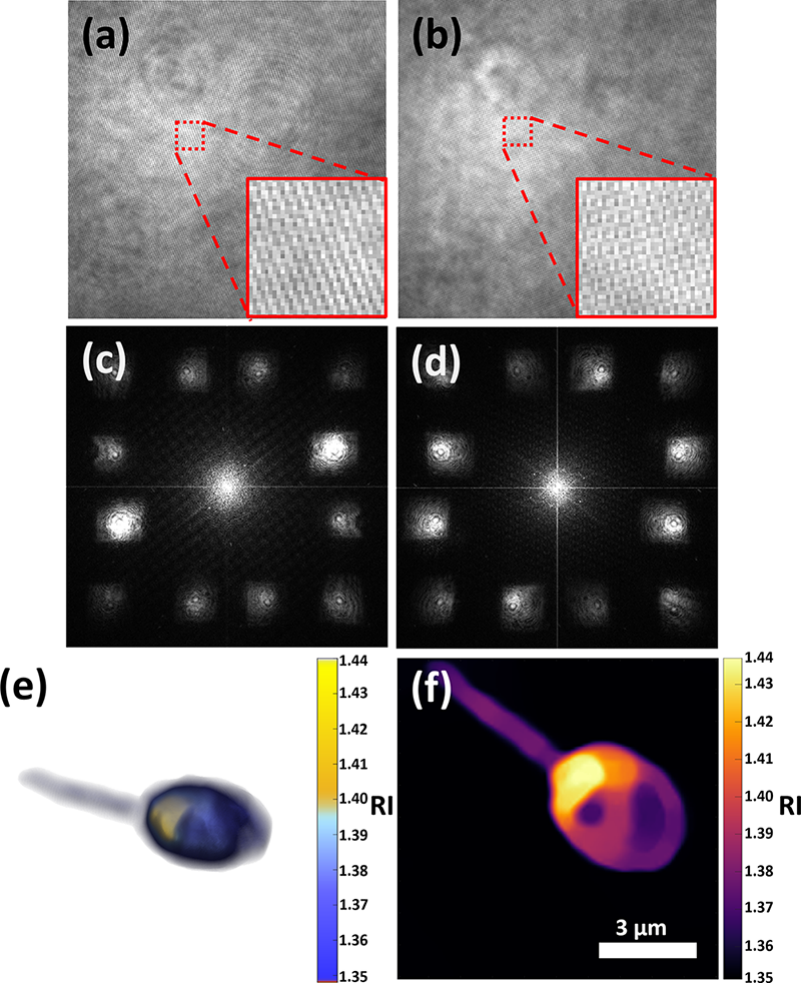
Double six-pack RI tomography results of the swimming human sperm cell from a single video frame. (a,b) Two six-pack multiplexed off-axis holograms from cameras C1 and C2, respectively, where (b) is shown after flipping horizontally. (c,d) Spatial frequency power spectra of (a,b), respectively. (e) Volume rendering of the 3D RI tomogram reconstructed from (a,b), shown at an oblique angle. (f) RI value map of (e) at the central plane. Scale bar of (f) also applies to (a,b).

A swimming sperm cell was then recorded. Figure 5a,b shows the synchronized holograms of a single video frame from cameras C1 and C2, and Figure 5c,d shows the spatial frequency power spectra of these holograms, respectively. Figure 5e shows a volume rendering of the reconstructed tomogram from the 12 CC terms at an oblique angle. It can be seen that the sperm cell head possesses a nucleus (yellow region, RI > 1.40) and acrosome (blue region, RI < 1.40). This RI threshold for the nucleus is based on the work of Habaza et al.,^30^ who found that for a 632.8 nm illumination wavelength, the nucleus possesses an RI of approximately 1.40. We take this as a reasonable lower limit for the more densely packed sperm cell nucleus. The midpiece is also visible, though the tail is mostly missing, due to being roughly equal in diameter to the diffraction-limited spot size and rapidly moving in different axial planes. Visualization 3 shows the video tomogram of this swimming cell at 7 fps. Figure 5f presents the RI of the cell from Figure 5e at the central plane using the perceptually uniform inferno color map. The nucleus and acrosome are clearly visible, and there also appears to be a vacuole, indicated by the circular dark region of low RI next to the nucleus. According to the World Health Organization criteria,^31^ a sperm such as this, with a vacuole in the nuclear region, should not be used for fertilization.

## Discussion and Conclusions

We presented a new technique for achieving dynamic scan-free optical tomography of rapidly moving biological cells with 12 times more data at each time point and demonstrated experimental results on flowing microbeads and a swimming human sperm cell. The technique is capable of producing tomograms of dynamic samples with accurate RI values and is not limited to sparse samples, though strongly birefringent or polarizing samples may cause artifacts due to the use of polarizers and polarized light. While the synchronization of the cameras was acceptable for the recorded samples, the software trigger used was only able to synchronize cameras to within 2 ms, which would introduce error when imaging faster processes. However, this can easily be solved by utilizing cameras with an external hardware trigger, which typically enables synchronization to within 2 μs. Splitting the dynamic range of the camera between the multiplexed holograms could possibly cause reduction in the signal to noise ratio, yet this effect is practically negligible for phase images of mostly transparent samples such as isolated biological cells in a watery medium.^32^

Numerous studies show that cell birefringence properties are associated with cell function and behavior,^33−36^ and acquiring dynamic 3D birefringence data could further enhance study in this field. Weakly birefringent samples, such as the sperm head, do not prevent reconstruction in our system. In the future, the system could potentially be modified in order to enable single-shot acquisition of 3D birefringence parameters. This could be accomplished by using only one set of six circularly polarized illumination beams and interfering these beams with the same reference beams on each camera but at orthogonal polarizations. Reconstruction of these two different 3D complex tomograms should enable the calculation of birefringence parameters.^37^

In the future, it is possible to take advantage of the large amount of data present in this double six-pack technique to increase the number of images acquired and thereby increase the reconstruction accuracy, at the expense of spatial bandwidth per image, by dividing each of the 12 CC terms into multiple subterms, as seen in ref (18). By utilizing our 12-fold increase in dynamic tomographic data in this manner, the double six-pack tomography technique outlined in this work is expected to lead to dynamic single-shot tomography at accuracies comparable to that of scanning tomography of static samples.

## Methods

### Optical System for Double Six-Pack Holography

**Figure 6:**
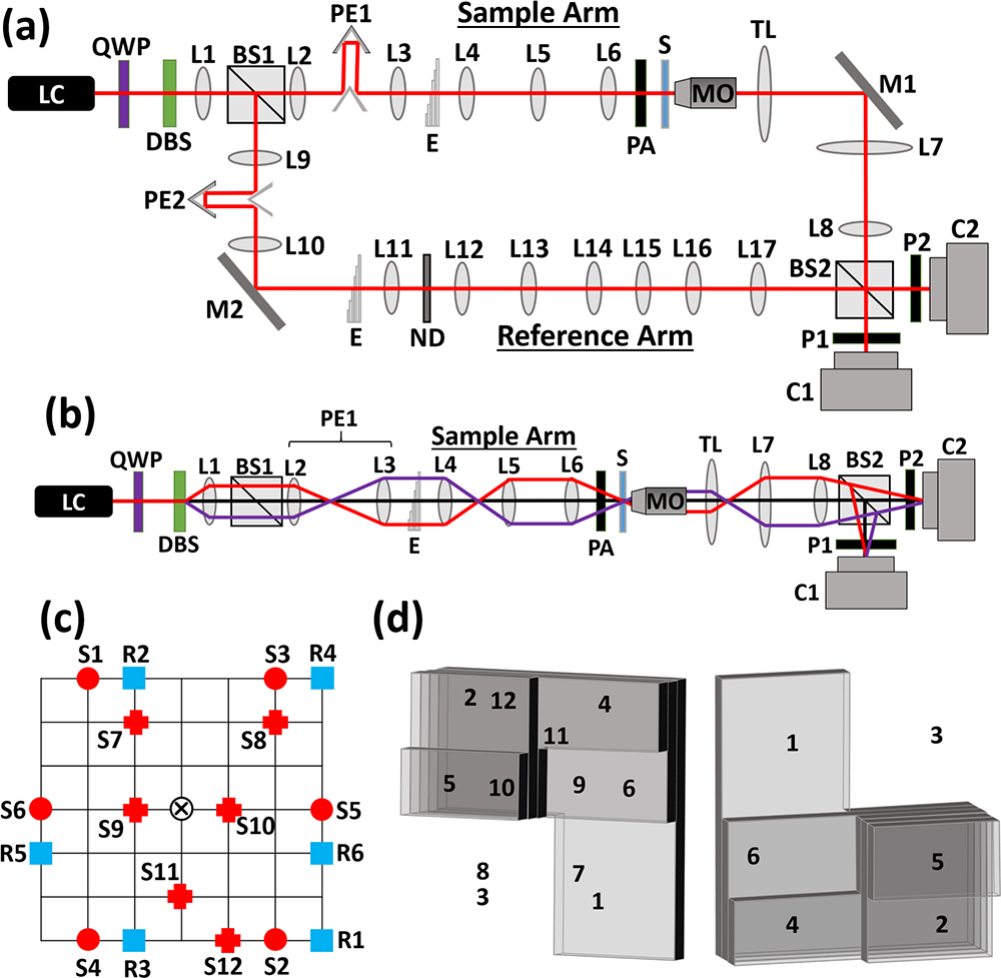
System diagram, illustration of the beam patterns and the echelon structures. (a) System diagram. The red line indicates the optical axis. LC, low-coherence light source; QWP, quarter waveplate; DBS, diffractive beam splitter; L1–L17, positive lenses; BS1–BS2, beam splitters; PE1 and PE2, periscopes; E, echelon; PA, polarizing array; S, sample; MO, microscope objective lens; TL, tube lens; M1 and M2, mirrors; P1 and P2, polarizers; C1 and C2, cameras; ND, neutral density filter. (b) Illustration of two sample beam paths (red and magenta lines). Mirrors of PE1 and M1 have been omitted for simplicity. (c) Sample beam positions at L6 and reference beam positions at L11. S1–S12, sample beams (marked in red); R1–R6, reference beams (marked in blue). Circles indicate first set of sample beams, and crosses indicate second set of sample beams. Each grid square is 2.8 mm × 2.8 mm for the sample beams and 1.4 mm × 1.4 mm for the reference beams. Optical axis is orthogonal to the image and is marked by ⊗. (d) Sample and reference echelon structures with beam positions by number. Left, sample arm echelon; right, reference arm echelon.

The proposed optical system is a modified Mach–Zehnder interferometer. Low coherence light with a wavelength of 632.8 nm is emitted by a supercontinuum laser source (NKT SuperK EXTREME) coupled to an acousto-optical filter (NKT SuperK SELECT) and a laser line filter (central wavelength: 632.8 nm, full width at half maximum: 3 nm), resulting in a laser source with a coherence length of 42.4 μm, collectively designated as LC in the system diagram shown in Figure 6a. Another light source of similar coherence length, such as a laser diode, may be used. However, it is critical that the coherence length be matched to the echelon optical path delays described later on. Additionally, if the coherence length is significantly shorter, the interference may not be present across the entire field of view.^38^ The LC light is then circularly polarized by a quarter waveplate, QWP, correspondingly aligned to the polarization axis of LC, and the circularly polarized beam is then split into a 7 × 11 pattern of collimated beams by a diffractive beam splitter, DBS (DigitalOptics Corporation). At this point, aside from the central on-axis beam, the beams diverge from the optical axis at various angles based on the pattern. Figure 6b shows an illustration of the beam paths of two sample beams. As the beams pass from lens to lens, they alternate from diverging and collimating to traveling in parallel to the optical axis and focusing. Next, the beams pass through lens L1 (focal length 50 mm) and 50:50 beam splitter BS1 and are split into the sample arm and the reference arm.

In the sample arm, the beams pass through L2 (focal length: 50 mm) and enter periscope PE1, composed of two mirrors with a 270° angle between their reflective faces, located on the axis, and two mirrors with a 90° angle between their reflective faces, located off-axis. The purpose of PE1 is to compensate for the added path delay introduced by periscope PE2, as will be explained later in this section. The beams are then passed to lens L3 (focal length 150 mm), with the ratio of focal lengths between L2 and L3 magnifying the beam pattern by a factor of 3, making it simpler to block all beams but the 12 desired sample beams shown in Figure 6c, designated as S1–S12. The beams then pass through an echelon, E, composed of sections of glass, 2 mm thick, illustrated in Figure 6d on the left. The echelon introduces a minimum optical path delay of 1.16 mm between the six pairs of two beams, such that beams passing through different sections cannot interfere with each other and produce undesired cross-talk, as this delay is greater than the coherence length of the source. The result is that each pair of sample beams may only interfere with one matching reference beam. The two sample beams in each pair are prevented from interfering with each other by assigning them orthogonal polarizations, as explained later on. The indicated positions of the beams in Figure 6c,d differ, as Figure 6c shows the sample beam pattern prior to lens L6 and not at the echelon E. Following this, the beams farthest from the optical axis, beams S1–S6 and S12, are given additional path delay by passing through a #1 glass slide (thickness 130–170 μm). This compensates for the additional path delay granted to the beams closer to the optical axis upon later passing through aspheric lens L6. Next, the 12 beams pass through lenses L4 (focal length 80 mm) and L5 (focal length 125 mm), which further magnify the pattern to be as large as possible in the *xy* plane, without being cut off by the aperture of the following lens L6 (aspheric, focal length 12.7 mm). The 12 sample beams then pass through L6 and illuminate the sample S from 12 different angles at a maximum off-axis angle of 39° in air, with distances from the optical axis at L6 shown in Figure 6c. Before illuminating the sample, the beams pass through an array of polarizers, PA, placed 6 mm before the sample, which polarizes beams S1–S6 at 0° and S7–S12 at 90° relative to each other, thereby allowing these two sets of sample beams to be separated when they later reach the cameras. In addition, since the light was circularly polarized until this point, all sample beams possess nearly identical intensities after the polarizers. While utilizing a higher NA lens for L6, such as an oil-immersion microscope objective lens, would gain higher illumination angles and increased tomogram resolution, placing the orthogonally polarizing PA before L6 results in sample-beam set polarizations losing orthogonality in the image plane due to the large change in angle, leading to cross-talk. Due to PA being placed between L6 and the sample, a relatively large focal length was necessary for L6, and oil or water immersion were made impractical. This may be resolved in the future by placing PA before L6 and calibrating each of the 12 polarizers according to final illumination angles of their beams such that the resulting polarizations of the sample-beam sets in the image plane will be orthogonal. After illuminating the sample, the sample is imaged by a microscope composed of microscope objective lens MO (Olympus UPlanSApo 60×/1.35 oil) and tube lens TL (focal length 150 mm). The image is then further magnified by lenses L7 (focal length 60 mm) and L8 (focal length 125 mm) and then split by 50:50 beam splitter BS2 to two identical cameras, C1 and C2 (Thorlabs DCC1545M, 8-bit monochromatic CMOS, 1280 × 1024 square pixels of 5.2 μm each). Polarizer P1 is aligned to 0° and P2 is aligned to 90°, thereby allowing only S1–S6 to reach C1 and only S7–S12 to reach C2. The final magnification of the system is 106×, with a hologram diffraction limited spot size of 469 nm.

In the reference arm, the beams travel similarly to the sample beams shown in Figure 6b. The reference beam pattern passes through lenses L9 and L10, which are identical to L2 and L3, respectively. On the way, the beams pass through periscope PE2, which is used to match the optical paths between the sample and reference arms. This is achieved by mounting the off-axis component of PE2 on a translation mount, allowing the distance traveled by the reflected beams to be precisely adjusted by moving the mirrors closer to or farther from the axis. Next, all beams but six are blocked, as illustrated in Figure 6c, and the six reference beams enter the reference arm echelon, shown on the right of Figure 6d. This echelon is different from the sample arm echelon as this specific positioning of the beams is necessary for the off-axis angles needed for six-pack holographic multiplexing. Following this, the beams pass through lenses L11–L16 (focal lengths of 75, 75, 100, 75, 50, and 50 mm, respectively). This is done in order to match the number of lenses used in the sample arm as well as to magnify the beam pattern for larger off-axis angles on the camera. The reference beams also pass through the neutral density filter, ND (optical density: 1.4), which matches the reference and sample beam intensities, and a 2 in. thick glass prism, which compensates for the large optical path delay introduced by the MO lens. While periscopes PE1 and PE2 could theoretically compensate for this path delay, expansion of the diverging beam pattern limited the maximum distance these periscopes could add without losing the beams at the edges of the pattern, and mirror diameter was limited by the proximity of the neighboring lenses and the necessity of maintaining 2f distance between them. This may be resolved in the future using a DBS with a smaller angle of divergence or by placing PE1 and PE2 at points in the system where beams travel in parallel, though the latter would typically require larger mirrors which may lead to complications. Finally, the reference beams pass through lens L17 (focal length 100 mm) and beam splitter BS2 to illuminate cameras C1 and C2 at six different off-axis angles. As the reference beams are still circularly polarized, the beams are equal in intensity after passing through polarizers P1 and P2 and are able to interfere with their six corresponding sample beams on both cameras. All lenses in the system were arranged in a telecentric (4f) configuration.

Prior to the experiment, C1 and C2 were registered by imaging a 1951 USAF target, where P1 and P2 were aligned to the same polarization angle. C2 was mounted on a translation and rotation mount with six degrees of freedom, allowing C2 to be registered precisely to C1, such that the images from both cameras were virtually identical once the image from C2 was flipped horizontally, with a minimum mean difference in value. Following this, upon loading the sample for the experiment and resetting the polarizers, a background hologram of an empty region of the sample was captured by both cameras. Next, videos of dynamic samples were captured by both cameras, with the camera acquisition synchronized using open-source μManager software.^39^ Videos were acquired at 7 fps and with an exposure time of 66 ms. While this exposure time would allow for frame rates of up to 15 fps, the software was unable to operate properly at higher frame rates than 7 fps. The software was able to synchronize the acquisition of each camera to within 2 ms or 3% of the exposure time used. In the future, hardware triggers can easily decrease the synchronization delay to only 2 μs. Taking the same 3% delay relative to the exposure time as the maximum allowable synchronization delay, the minimum exposure time would then be 66 μs. Given the diffraction-limited spot size of 469 nm, this would result in a maximum flow speed of approximately 7 mm/s, as limited by the camera synchronization, while avoiding motion blur, with the only limitation being sufficient illumination intensity.

**Figure 7:**
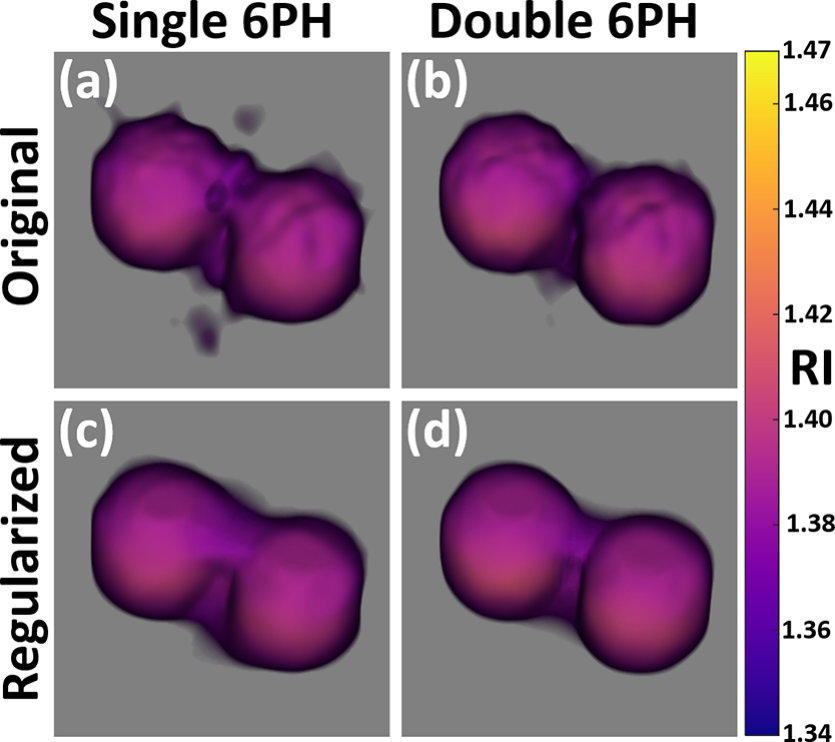
Single and double six-pack tomograms of the bead pair. (a) Original single six-pack tomogram. (b) Original double six-pack tomogram. (c) Regularized single six-pack tomogram. (d) Regularized double six-pack tomogram.

### RI Tomogram Reconstruction Process

The video frames from C2 were flipped horizontally, and each frame of the dynamic six-pack holography videos acquired by each camera was then 2D Fourier transformed and the six selected CC terms were cropped from each spatial frequency domain for a total of 12 terms. The 12 terms were then inverse Fourier transformed to produce 12 complex wavefront images, each from a different perspective of the sample. The complex wavefront images acquired from each video frame were then pixel-wise divided by the single background complex wavefront captured previously to compensate for phase curvature, and the phase was extracted and unwrapped.

Next, the 12 unwrapped phase images were median-filtered to remove noise and then processed by using the optical diffraction tomography algorithm^26^ under the Rytov assumption in order to reconstruct 3D RI tomograms for each frame. In this algorithm, the 2D spatial frequencies of each image were each mapped into a different 3D hemispheric surface, known as an Ewald cap. The Ewald caps were aligned in the spatial frequency plane based on their respective illumination angles, and their values were averaged where they overlapped, resulting in an Ewald sphere, a 3D spatial frequency distribution. An inverse Fourier transform was then applied, and the 3D RI distribution was then calculated by
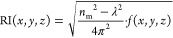
where *n*_m_ is the refractive index of the medium, λ is the illumination wavelength, and *f*(*x*,*y*,*z*) is the inverse Fourier transform of the Ewald sphere.

Following this, an iterative non-negativity algorithm^40^ was applied to correct RI values lower than the RI of the medium and improve reconstruction accuracy. This process involved setting all values in the reconstructed 3D RI distribution that were lower than n_m_ to be equal to n_m_. Then, a Fourier transform was performed, and we located the voxels that were previously valued at zero in the Fourier transform but were now nonzero. These voxels were allowed to remain nonzero, and the voxels originally containing nonzero values, i.e., the initial data, which had also been altered by this process, were reset to their initial values. This process was then iterated until accurate reconstruction was achieved. Finally, a total variation regularization algorithm^41^ was utilized in order to further remove noise and decrease blurring. This algorithm took the approximated point spread function of the system as the blurring operator and implemented Nesterov’s iterative optimal first-order method for solving μ-strongly convex problems while using the L-Lipschitz continuous gradient.^41^ The algorithm used was acquired from ref (42).

**Figure 8:**
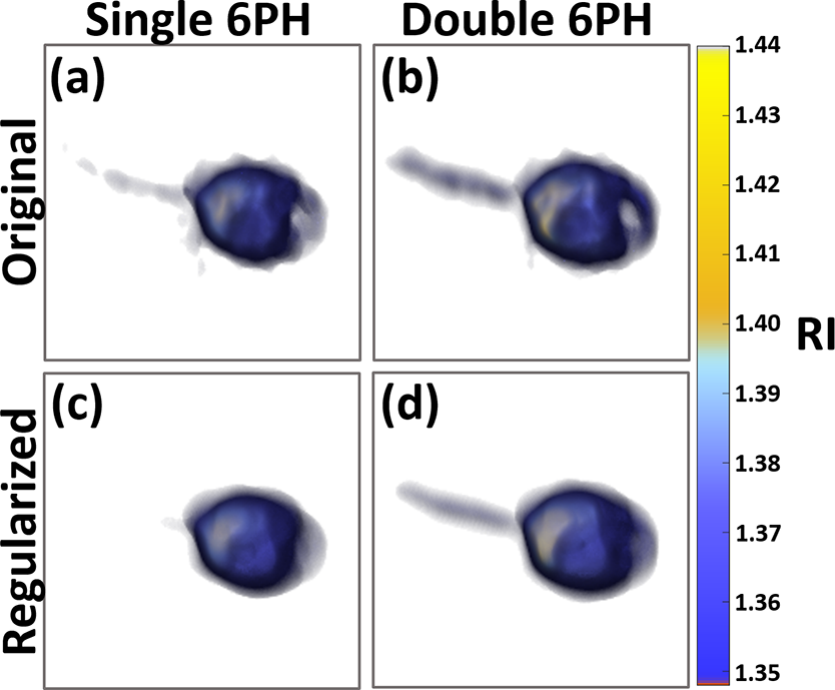
Single and double six-pack tomograms of the sperm cell. (a) Original single six-pack tomogram. (b) Original double six-pack tomogram. (c) Regularized single six-pack tomogram. (d) Regularized double six-pack tomogram.

In order to assess the quality of the double six-pack reconstruction versus the single six-pack and the effects of the regularization algorithm on the final tomogram, we reconstructed the 3D RI tomograms of the pair of beads and the sperm cell, shown in Figures 3 and 5, respectively, using only the single six-pack hologram from sample beams S1–S6 in addition to the double six-pack tomograms shown in those figures. Figure 7 shows volumetric images of the single and double six-pack tomograms before and after the regularization. In comparison to the non-regularized single six-pack tomogram shown in Figure 7a, the nonregularized double six-pack tomogram in Figure 7b results in a double six-pack tomogram which better reconstructs the entire 3D structure of the bead pair as the region between the beads has RI values closer to that of the medium and the beads are almost fully separated in the double six-pack tomogram. This is in contrast to the single six-pack tomogram in which higher erroneous RI values appear between the beads. However, prior to the regularization in Figure 7c,d, it can be seen that there is a significant presence of noise leading to an irregular surface for the beads. After the regularization, the surface is rounder and smoother in both Figure 7c,d as the noise has been reduced. Additionally, the average RI values of the beads in Figure 7a,b relative to those in Figure 7c,d, respectively, are virtually identical, with a difference of less than 0.1%. However, the RI values between the beads increase in both cases, resulting in decreased RI accuracy in this region. In the case of the sperm cell tomograms, it can be seen that the single six-pack tomogram in Figure 8a, in comparison to the double six-pack tomogram in Figure 8b, has lower RI values for the nucleus and does not reconstruct the midpiece effectively. In Figure 8c, we can see that the regularization of the six-pack tomogram further reduces the presence of the midpiece while removing noise, almost entirely erasing it. However, while in Figure 8d we see a similar effect, the tomogram still possesses a visible midpiece and acrosome in addition to decreased noise.

## Supporting Information Available

Visualization 1: Rotating view of bead 3D tomogram (MP4)
Visualization 2: Dynamic video tomogram of flowing beads (MP4)
Visualization 3: Dynamic video tomogram of a swimming sperm cell (MP4)

## Notes

Ethics Declaration: the human sperm study was approved by Tel Aviv University’s institutional review board.

## Abbreviations

2D: two-dimensional
3D: three-dimensional
RI: refractive index
CC: cross-correlation
DC: autocorrelation
6PH: six-pack holography
